# Biosynthesis of vanillin glucoside in *Escherichia coli*

**DOI:** 10.1016/j.synbio.2026.08.008

**Published:** 2026-09-08

**Authors:** Mingyu Tian, Jinyi Li, Yong Du, Xiaolin Shen, Jia Wang, Xinxiao Sun, Qipeng Yuan

**Affiliations:** State Key Laboratory of Chemical Resource Engineering, Beijing University of Chemical Technology, Beijing, 100029, China

**Keywords:** Vanillin, Vanillyl alcohol, Vanillin glucoside, *De novo* biosynthesis, SAM cycle, *Escherichia coli*

## Abstract

Vanillin is a widely used flavor compound in the food, cosmetic, and pharmaceutical industries. Although microbial biosynthesis offers a green alternative to conventional chemical synthesis, challenges such as unstable heterologous enzyme expression, insufficient methyl donor supply, and product cytotoxicity limit *de novo* production. Here, we engineered *Escherichia coli* to produce vanillyl alcohol, vanillin, and vanillin glucoside directly from glycerol. Genomic integration of the alcohol dehydrogenase *ADH6* and multi-copy of the caffeic acid *O*-methyltransferase COMT gene eliminated the need for plasmid-based expression, yielding 1.09 g/L vanillyl alcohol in shake flasks. To overcome methylation bottlenecks, we reinforced the *S*-adenosyl-l-methionine (SAM) cycle by enhancing SAM biosynthesis, accelerating *S*-adenosyl-l-homocysteine (SAH) hydrolysis, and establishing methionine regeneration, which increased vanillyl alcohol production to 1.95 g/L. Subsequent deletion of four endogenous aldehyde-reduction genes together with removal of heterologous *ADH6* enabled vanillin accumulation at 551.6 mg/L. Introduction of the glucosyltransferase UGT72E3/2 converted vanillin into vanillin glucoside, reaching 1.37 g/L in shake flasks and 2.12 g/L in a 3-L fed-batch fermenter. This work establishes a modular platform for efficient production of vanillin and its derivatives in *E. coli*.

## 1 Introduction

Vanillin and vanillyl alcohol are highly valuable aromatic compounds that are widely found in *Vanilla planifolia* and the medicinal plant *Gastrodia elata* [1,2]. Vanillyl alcohol is not only a key precursor for vanillin biosynthesis, but also exhibits notable anti-inflammatory and antioxidant activities [3,4]. Vanillin, the principal component responsible for vanilla flavor, is one of the most important flavoring agents worldwide and is extensively used in the food, fragrance, pharmaceutical, and other emerging industries [5]. At present, the global demand for vanillin has reached 20000 tons annually; however, approximately 90% of vanillin is produced through petrochemical synthesis, while the annual output of vanillin extracted from natural plants remains below 2000 tons [6] In recent years, growing consumer demand for natural products has driven the development of vanillin biosynthesis technologies, and some biosynthetically produced nature-identical vanillin has been approved for use as a food additive by major food safety regulatory authorities worldwide [7]. Although chemical synthesis from petrochemical-derived precursors such as guaiacol can meet industrial demand at relatively low cost, it is less compatible with the growing preference for natural-label products [[8], [9], [10]]. In contrast, direct extraction from vanilla pods is constrained by long cultivation cycles, limited plant resources, low yield, and high production costs [[11], [12], [13]]. Therefore, microbial biosynthesis using renewable carbon sources has emerged as a promising green and sustainable alternative for producing vanillin and its derivatives.

Through engineering of the endogenous shikimate pathway in microorganisms, the biosynthesis of vanillyl alcohol and vanillin has been achieved in multiple chassis strains [14,15] Nevertheless, the production titers reported to date differ substantially across species, substrates, and fermentation modes, making it difficult to directly assess the competitiveness of individual studies. To facilitate a systematic comparison and to better position the current work among the state-of-the-art, we compiled the representative titers of vanillin, vanillin glucoside, and vanillyl alcohol in various microbial hosts in (Table 1).

**Table 1:** Comparison of vanillin, vanillin glucoside, and vanillyl alcohol production titers in various microbial hosts.

Product	Host	Output	Development way	Substrate Precursor	Reference
Vanillin	*C. glutamicum*	22 g/L	Batch fermentation	Vanillic acid	[16]
	*P. putida*	2.85 g/L 4.47 g/L	Shake flask In-situ extraction	Ferulic acid	[17]
	*P. putida*	16.1 g/L	Resting cell	Isoeugenol	[18]
	*K. phaffii*	1.05 g/L	Bioreactor	Glucose Caffeic acid	[19]
	*E. coli*	0.481 g/L	5-L fermenter	Glucose	[20]
	*B. subtilis*	0.152 g/L	Shake flask	Shikimic acid	[21]
	*S. cerevisiae*	0.533 g/L	Shake flask	Glucose	[22]
Vanillin glucoside	*S. cerevisiae*	7.476g	5-L fermenter	Glucose	[22]
Vanillyl alcohol	*E. coli*	0.24 g/L	Shake flask	Glucose	[23]
	*E. coli*	0.499 g/L	Shake flask	3,4-dihydroxybenzyl alcohol	[23]
	*E. coli*	0.559 g/L 3.89 g/L	Shake flask 5-L fermenter	Glycerol	[24]

Despite these advances, existing studies still face multiple challenges, including limited methylation efficiency, by-product accumulation caused by endogenous reductases, and inhibition of cell growth by product toxicity. Among these limitations, COMT-mediated methylation is considered the most critical rate-limiting step. Its efficiency depends not only on the catalytic activity of the enzyme itself, but also on intracellular SAM availability and feedback inhibition caused by the by-product *S*-adenosyl-l-homocysteine (SAH) [25]. To address these issues, several strategies have been developed to improve methylation efficiency, including optimization of SAM biosynthesis and acceleration of SAH degradation through the expression of SAH hydrolase. Tan et al. increased SAM production to 2.46 g/L in *S. cerevisiae* through multi-module metabolic engineering [26]. Luo et al. enhanced methyl anthranilate production in *Corynebacterium glutamicum* by 69% through the expression of *sahH* to promote SAH hydrolysis [27]. Zhang et al. further constructed a universal methylation platform based on a triple SAM cycle, which significantly improved the biosynthetic efficiency of multiple methylated products, including vanillyl alcohol [28]. The methylation efficiency was improved by using the laboratory-preserved from *Arabidopsis thaliana* O-methyltransferase mutant AtCOMT-V314I. Therefore, systematically improving methylation efficiency, reducing vanillin reduction, and alleviating product toxicity remain central challenges for the efficient biosynthesis of vanillin and its derivatives.

**Fig. 1:**
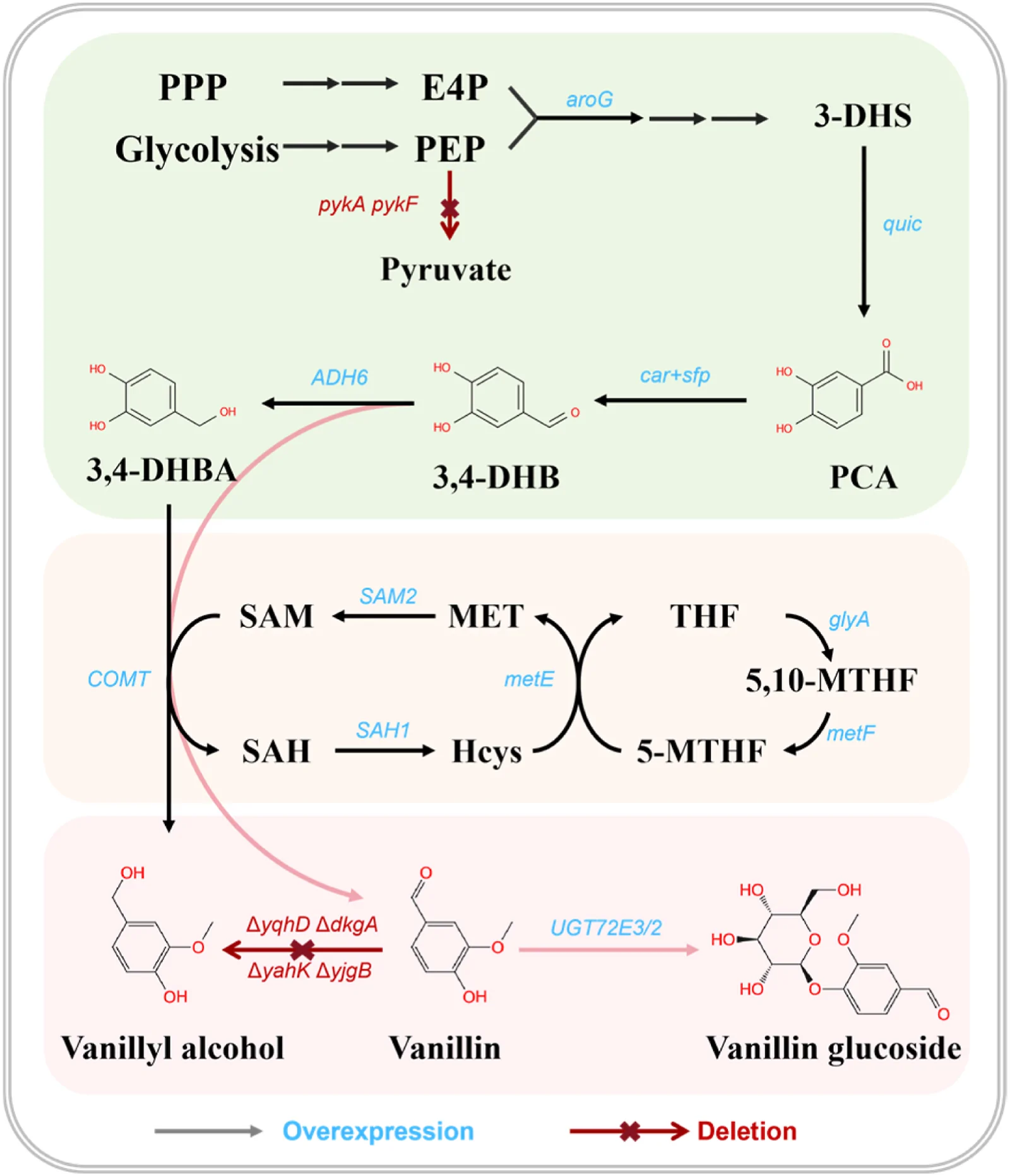
*De novo* biosynthesis of vanillyl alcohol, vanillin and vanillin glucoside. Gene deletions are marked in red, overexpression of genes in blue. *pykA/F*, encoding pyruvate kinases; *aroG*, encoding feedback-inhibition-resistant 2-dehydro-3-deoxyphosphoheptonate aldolase; *quiC*, encoding 3-dehydroshikimate dehydratase; car, encoding carboxylic acid reductase; *sfp*, encoding phosphopantetheinyl transferase; *ADH6*, encoding alcohol dehydrogenase; *COMT*, encoding the caffeate O-methyltransferase; *SAM2*, encoding the *S*-adenosylmethionine synthase 2; *glyA*, encoding the serine hydroxymethyltransferase; *metE*, encoding the 5-methyltetrahydropteroyltriglutamate-homocysteine methyltransferase; *metF*, encoding the 5,10-methylenetetrahydrofolate reductase; *SAH1*, encoding the adenosylhomocysteinase; *yqhD*, encoding the NADPH-dependent aldehyde reductase; *dkgA*, encoding the methylglyoxal reductase; *yahK*, encoding the aldehyde reductase; *yjgB*, encoding the aldehyde reductase; *UGT72E3/2*, encoding the UDP-glycosyltransferase.

Based on these considerations, this study aimed to construct a stable and efficient *Escherichia coli* platform for the *de novo* biosynthesis of vanillyl alcohol, vanillin, and vanillin glucoside. To reduce plasmid-associated burden and improve pathway stability, key pathway genes, including *ADH6* and *COMT*, were integrated into the genome to establish a vanillyl alcohol-producing chassis. To address the methylation bottleneck, an intracellular methylation-enhancing module was further constructed by reinforcing SAM biosynthesis, promoting SAH hydrolysis, and strengthening methionine-cycle regeneration, which substantially improved vanillyl alcohol production. Subsequently, endogenous reductase genes responsible for the conversion of vanillin to vanillyl alcohol were deleted to enable direct vanillin accumulation. Finally, a specific vanillin glucosyltransferase was identified and introduced to expand the pathway toward vanillin glucoside biosynthesis. Overall, this work integrates genome-based pathway stabilization, methyl-cycle reinforcement, reductive-branch blockade, and product glycosylation, providing a systematic engineering strategy for the biosynthesis of methylated aromatic compounds and their glycosylated derivatives (Fig. 1).

## 2 Materials and methods

### 2.1 Strains and plasmids

All strains and plasmids used in this study are listed in Supplementary Table S1. *E. coli* Trans5α was used for plasmid construction and propagation, while *E. coli* BW25113 and its derivative strains were used as chassis strains for the biosynthesis of target products. The plasmids pZE12-luc, pCS27, and pSA74 were used for metabolic pathway assembly, whereas pCas9 and pTarget were employed for genome integration.

### 2.2 Gene manipulation

Genome-integrated strains were constructed using CRISPR/Cas9-mediated genome editing, with integration performed at previously reported pseudogene loci [29,30]. PCR amplification and homologous recombination for plasmid construction were carried out according to the manufacturers’ standard protocols. The plasmids was constructed by using the Seamless Cloning and Assembly Kit (CU201, TransGen Biotech, China). Successfully constructed plasmids in *E. coli* Trans1 were subsequently introduced into the corresponding chassis strains by electroporation.

### 2.3 Culture conditions

For *de novo* biosynthesis experiments, a single colony was inoculated into 4 mL LB (5 g/L yeast extract, 10 g/L sodium chloride, and 10 g/L tryptone) medium supplemented with the appropriate antibiotics and cultivated at 37°C for 16 h. Subsequently, 1 mL of the overnight seed culture was transferred into 50 mL M9Y (20 g/L glycerol, 6 g/L yeast extract, 6.78 g/L Na_2_HPO_4_, 3 g/L KH_2_PO_4_, 0.5 g/L NaCl, 1 g/L NH_4_Cl, 1 mM MgSO_4_, 0.1 mM CaCl_2_, and 2 g/L methionine) medium and incubated at 37°C with shaking. When the optical density at 600 nm (OD_600_) reached 0.6, IPTG was added to a final concentration of 0.5 mM for induction, after which the culture temperature was shifted to 33°C for continued cultivation.

For substrate-feeding experiments, a single colony was inoculated into 4 mL LB medium containing the appropriate antibiotics and cultivated at 37°C for 12 h. Then, 1 mL of the overnight seed culture was transferred into 50 mL M9Y medium and incubated at 37°C with shaking. When the OD_600_ reached 0.6, IPTG was added to a final concentration of 0.5 mM for induction. Four hours after induction, the corresponding substrate was added to a final concentration of 500 mg/L. Samples were collected after 6 h of reaction and analyzed by high performance liquid chromatography (HPLC).

For fed-batch fermentation, strain TMY29 was inoculated into a 3-L bioreactor containing 1 L of fermentation medium. The initial medium consisted of 10 g/L glycerol, 10 g/L yeast extract, 5 g/L peptone, 2 g/L (NH_4_)_2_SO_4_, 4 g/L KH_2_PO_4_, 2 g/L Na_2_HPO_4_, 1.5 g/L MgSO_4_, and 0.1 g/L CaCl_2_. After inoculation, the culture was maintained at 33°C. When the OD_600_ reached approximately 6, IPTG was added to a final concentration of 0.5 mM for induction. The feeding solution was divided into two reservoirs: reservoir A contained 500 g/L glycerol, and reservoir B contained 50 g/L methionine. The feeding rates were set at 4 mL/h for reservoir A and 2 mL/h for reservoir B. During fermentation, the pH was maintained at 7.0. Cell growth was monitored by measuring OD_600_, and the fermentation supernatant was collected for metabolite analysis by HPLC.

### 2.4 High performance liquid chromatography

Both standards and samples were quantified by HPLC equipped with a reversed-phase Diamonsil C18 column and a UV–visible detector. Mobile phase A consisted of water containing 0.1% formic acid, and mobile phase B was pure methanol. The column temperature was maintained at 40°C, and the flow rate was set at 0.8 mL/min. The following gradient program was used: 0–20 min, mobile phase B was increased from 5% to 50%; 20–22 min, mobile phase B was maintained at 100%; 22–24 min, mobile phase B was decreased from 100% to 5%; and 24–29 min, mobile phase B was maintained at 5%. Quantification was performed based on the peak areas detected at 280 nm.

## 3 Results

### 3.1 Genomic integration of *ADH6* and multi-copy of *COMT* for stable vanillyl alcohol production

**Fig. 2:**
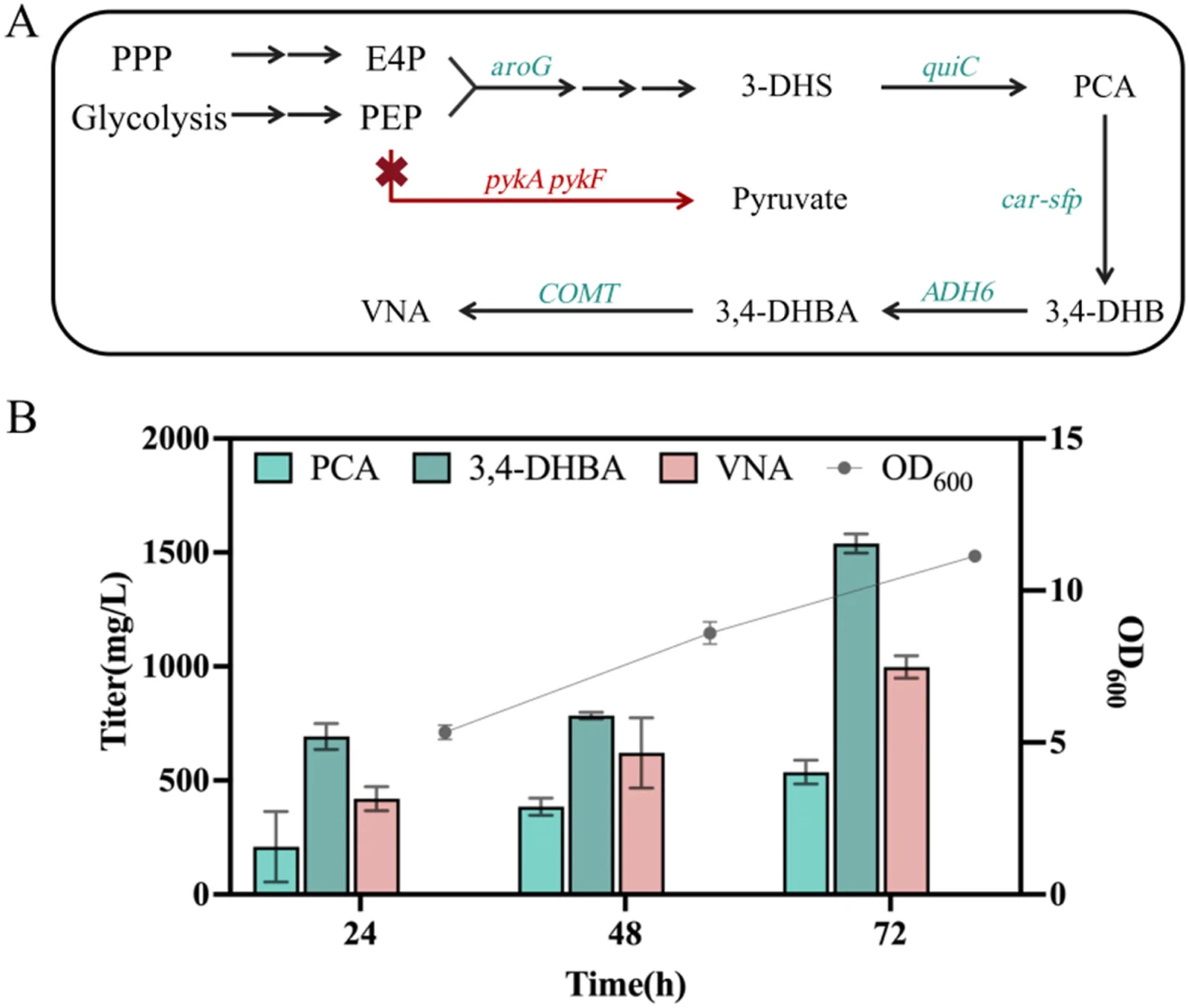
*De novo* biosynthesis of vanillyl acohol in *E. coli*. (A) Metabolic pathway for *de novo* biosynthesis of vanillyl alcohol; (B) *De novo* biosynthesis of vanillyl alcohol by strain TMY01.

In our previous work, *de novo* biosynthesis of vanillyl alcohol was achieved using an *E. coli* co-culture system [24]. To simplify the production system and evaluate whether vanillyl alcohol could be efficiently produced in a single strain, we used Lab02, a PCA-producing chassis strain previously constructed in our laboratory, as the starting strain for pathway reconstruction. In Lab02, the endogenous shikimate pathway had been engineered to enhance the supply of PCA, a key aromatic precursor for vanillyl alcohol biosynthesis (Fig. 2A). The downstream pathway modules responsible for PCA reduction, aldehyde reduction, and *O*-methylation were then introduced into Lab02 using the plasmids pCS-car-sfp, pSA-*ADH6*, and pZE-AtCOMT-V314I, respectively, generating the vanillyl alcohol-producing strain TMY01. After 72 h of fermentation in M9Y medium supplemented with 2 g/L methionine, TMY01 produced 997.63 mg/L vanillyl alcohol (Fig. 2B). These results demonstrated that *de novo* biosynthesis of vanillyl alcohol could be efficiently achieved in a single *E. coli* strain, providing a basis for subsequent pathway stabilization and methylation optimization.

**Fig. 3:**
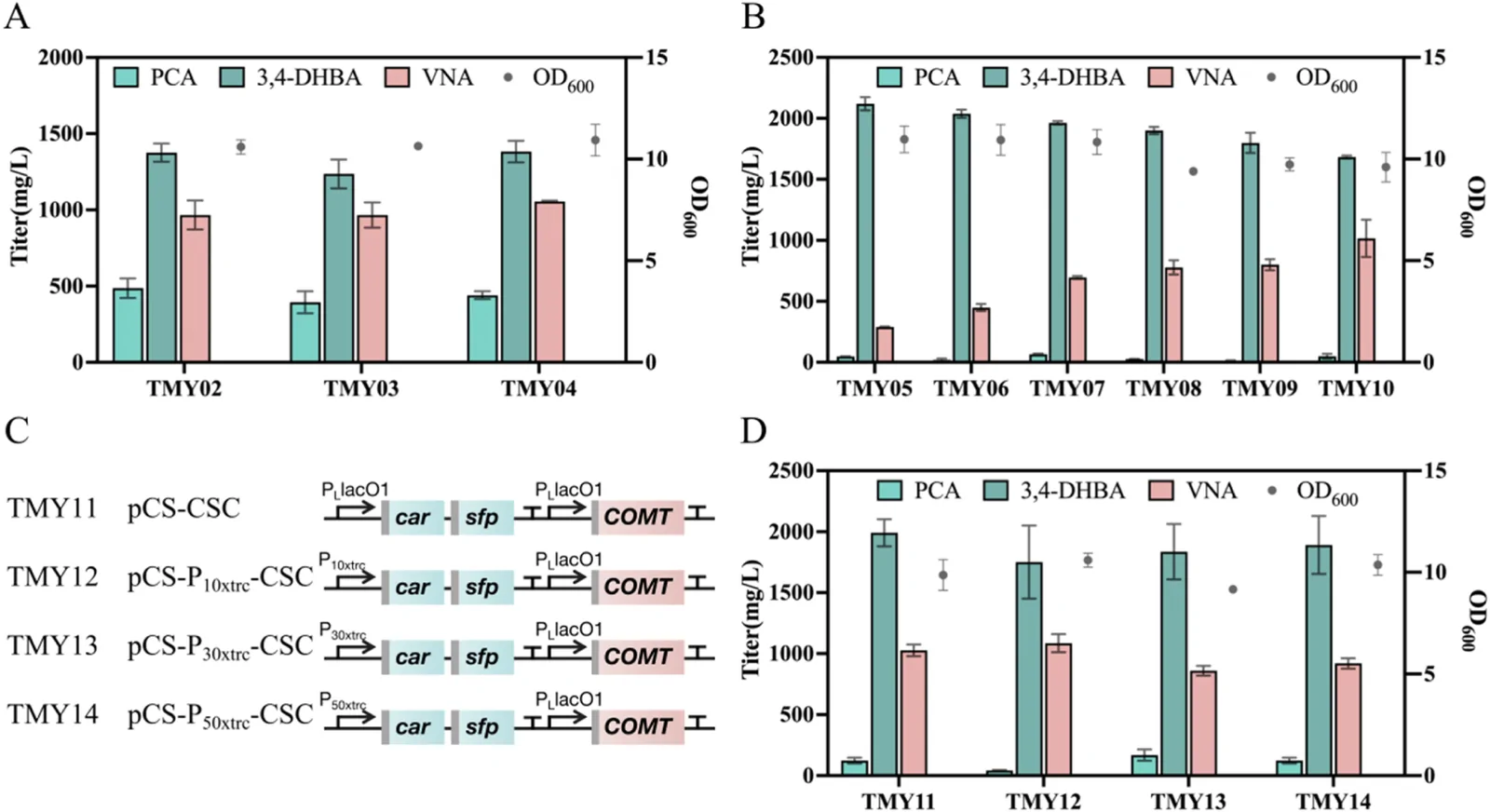
Effect of multi-copy integration on vanillyl alcohol biosynthesis. (A) Integration of *ADH6* gene driven by promoters with different strengths; (B) Integration of 6 copies of AtCOMT-V314I gene; (C) Plasmid construction using promoters with different strengths; (D) Vanillyl alcohol production via car-sfp expression driven by promoters with different strengths.

To improve pathway stability and reduce the metabolic burden associated with plasmid maintenance, the key reductase gene *ADH6* was integrated into the genome of the PCA-producing chassis strain Lab02. To evaluate the effect of *ADH6* expression level on vanillyl alcohol production, *ADH6* was placed under the control of three constitutive promoters with different strengths, namely P10trc, Plpp2.0, and Plpp4.0. The resulting genome-integrated chassis strains were subsequently transformed with pZE-AtCOMT-V314I and pCS-car-sfp, generating the vanillyl alcohol-producing strains TMY02, TMY03, and TMY04, respectively. Fermentation results showed that TMY04, in which *ADH6* was driven by the strongest promoter Plpp4.0, exhibited the best performance, producing 1.05 g/L vanillyl alcohol (Fig. 3A). This result indicates that sufficient *ADH6* expression is important for the efficient conversion of protocatechualdehyde (3,4-DHB) to 3,4-DHBA, and that genomic integration of *ADH6* can maintain efficient pathway flux while reducing dependence on plasmid-based expression.

In order to further reduce the plasmid dependence and improve the stability of the pathway, we gradually integrated the AtCOMT-V314I expression cassette driven by Plpp4.0 into the genome on the basis of the host strain integrated with *ADH6*, and obtained engineering strains carrying one to six copies of *AtCOMT*-V314I, respectively. In order to facilitate the distinction, these strains were named TMY05, TMY06, TMY07, TMY08, TMY09 and TMY10. Among them, TMY05 to TMY10 corresponded to one, two, three, four, five and six copies of AtCOMT-V314 I integrated strains, respectively. All strains only carried pCS-car-sfp plasmid for subsequent transformation. Fermentation analysis showed that replacing plasmid-based AtCOMT-V314I expression with genome-integrated expression markedly reduced PCA accumulation to approximately 48.12 mg/L, likely because removal of the COMT-expressing plasmid alleviated plasmid-associated metabolic burden and improved pathway balance. Meanwhile, vanillyl alcohol production gradually increased with increasing AtCOMT-V314I copy number. The six-copy integrated strains TMY10 produced 1.02 g/L vanillyl alcohol, which was comparable to the titer obtained with the original plasmid-based *COMT* expression system (Fig. 3B). These results indicate that multi-copy genomic integration of *AtCOMT*-V314I could effectively replace plasmid-based *COMT* expression, allowing one plasmid to be removed while maintaining comparable vanillyl alcohol production. Therefore, integration of both *ADH6* and *AtCOMT*-V314I reduced plasmid dependence without compromising production performance.

To further examine whether methyltransferase expression remained limiting, the *COMT* gene driven by the PLlacO1 promoter was inserted into the second open reading frame of pCS-car-sfp, generating pCS-CSC. Replacement of pCS-car-sfp in TMY10 with pCS-CSC yielded strain TMY11. However, additional plasmid-based expression of *COMT* in the six-copy integrated strains did not markedly improve vanillyl alcohol production, which reached 1.03 g/L (Fig. 3D). This result suggests that simply increasing *COMT* dosage was insufficient to further enhance vanillyl alcohol biosynthesis.

We next optimized the expression of CAR to improve the balance between precursor conversion and downstream methylation. The P_L_lacO1 promoter controlling the first open reading frame of pCS-CSC was replaced with promoters of different strengths, including P10trc, P30trc and P50trc. The resulting plasmids were introduced into the chassis strain carrying six-copy integrated of the methyltransferase cassette, generating strains TMY12, TMY13, and TMY14, respectively (Fig. 3C). Fermentation analysis showed that the strain carrying the P10trc-driven CAR module exhibited the best performance, producing 1.09 g/L vanillyl alcohol, which was higher than the titers obtained with the other promoter variants (Fig. 3D). In this strain, PCA accumulation was reduced to 42.6 mg/L. The superior performance of the P10trc promoter may be attributed to a more appropriate balance between CAR-mediated precursor conversion and the metabolic burden imposed by heterologous pathway expression. Together, these results indicate that after genomic integration of *ADH6* and *AtCOMT*-V314I, further improvement of vanillyl alcohol production required not only balanced pathway enzyme expression but also optimization of methyl-donor supply and recycling.

### 3.2 Reinforcement of the SAM cycle to enhance methylation efficiency

**Fig. 4:**
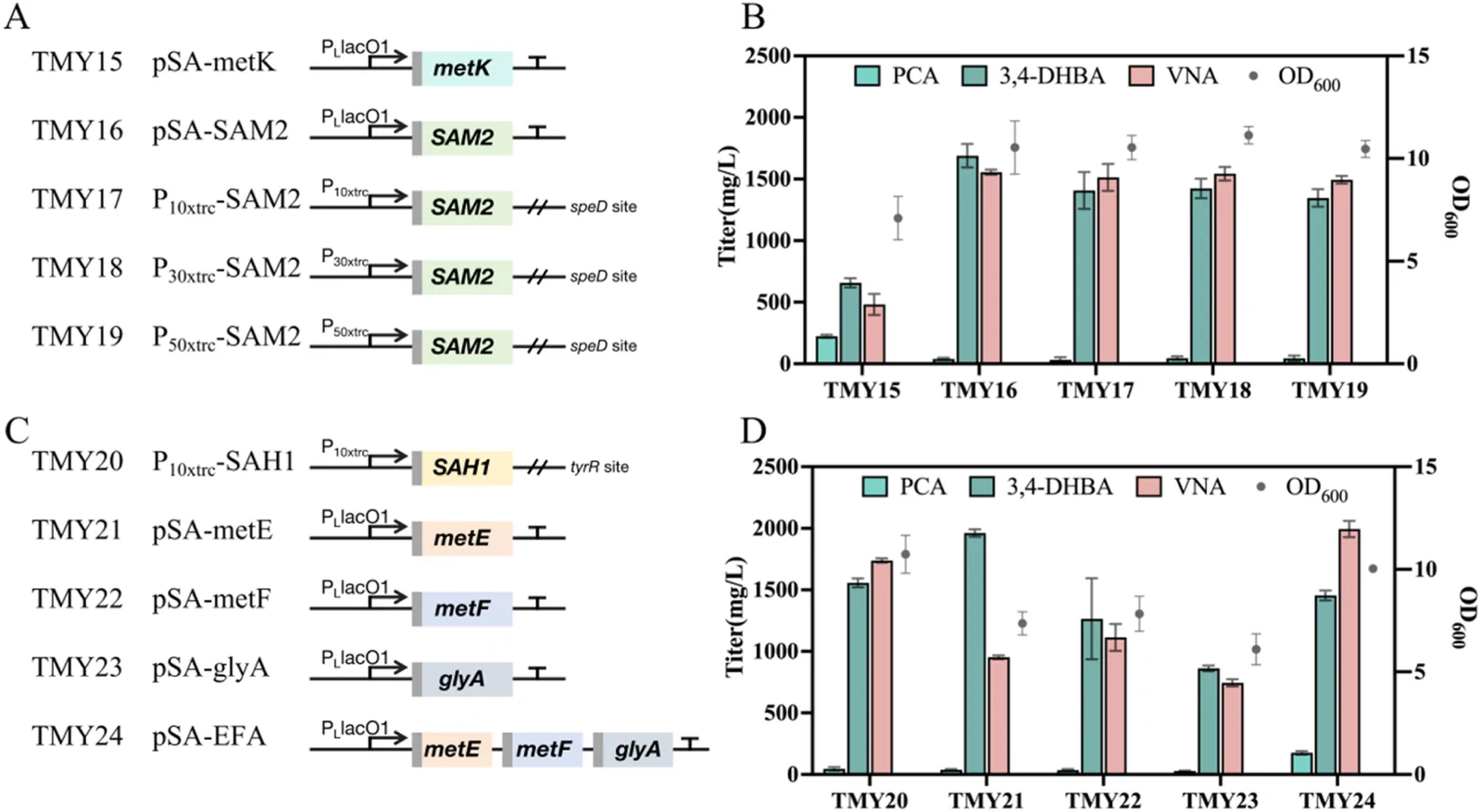
Improved methylation efficiency via enhanced SAM supply and cycle. (A) Plasmid construction of SAM synthetases from different sources and genomic integration of *SAM2*; (B) Effect of enhanced SAM supply on vanillyl alcohol production; (C) Enhanced SAH hydrolysis to relieve feedback inhibition on methyltransferase; (D) Enhanced methionine regeneration for SAM cycle optimization.

Further modulation of pathway enzyme expression did not markedly improve vanillyl alcohol production, suggesting that factors beyond pathway enzyme abundance might limit the methylation reaction. Since methionine had already been supplemented in the fermentation medium, we reasoned that the conversion of methionine to SAM, catalyzed by methionine adenosyltransferase, might restrict intracellular methyl donor availability [31] Previous studies have shown that overexpression of the endogenous *E. coli* SAM synthetase gene *metK* or the *Saccharomyces cerevisiae* SAM synthetase gene *SAM2* can enhance SAM biosynthesis, with *SAM2* showing particular potential because it is naturally insensitive to SAM-mediated feedback inhibition [26,32,33]. Therefore, we compared the effects of strengthening SAM biosynthesis using *metK* and *SAM2*. Plasmids pSA-metK and pSA-SAM2 were constructed and introduced into TMY12, generating strains TMY15 and TMY16, respectively. Fermentation analysis showed that overexpression of endogenous metK impaired cell growth and reduced vanillyl alcohol production to 481.9 mg/L. In contrast, expression of heterologous *SAM2* maintained robust cell growth and increased vanillyl alcohol production to 1.56 g/L (Fig. 4A). These results suggest that *SAM2* was more suitable than *metK* for reinforcing SAM biosynthesis in this production system, likely because it improved methyl donor supply while avoiding excessive metabolic burden or feedback regulation.

To further reduce plasmid dependence, *SAM2* was subsequently integrated into the genomic *speD* locus, whose deletion has been reported to enhance SAM availability [34]. SAM2 was placed under the control of three promoters with different strengths, P10trc, P30trc, and P50trc, generating strains TMY17, TMY18, and TMY19, respectively. Fermentation results showed that genomic integration of *SAM2* effectively supported vanillyl alcohol production, with titers reaching 1.51 g/L, 1.54 g/L, and 1.49 g/L, respectively (Fig. 4B). The comparable titers obtained with different promoter strengths indicate that chromosomal expression of *SAM2* was sufficient to enhance methylation-related production without introducing an additional plasmid. Therefore, genome-integrated *SAM2* provided a more stable strategy for reinforcing SAM biosynthesis in the vanillyl alcohol-producing chassis.

During COMT-mediated methylation, SAM is converted into *S*-adenosyl-l-homocysteine (SAH), which can inhibit many SAM-dependent methyltransferases [35]. Therefore, in addition to strengthening *de novo* SAM biosynthesis, promoting SAH turnover and methionine regeneration may provide a more effective strategy for sustaining SAM-dependent methylation [36,37]. To enhance SAH hydrolysis, the *SAH1* gene from *Saccharomyces cerevisiae*, encoding *S*-adenosyl-l-homocysteine hydrolase, was integrated into the genomic *tyrR* locus of TMY18 under the control of the P10trc promoter, generating strain TMY20. Since SAH hydrolysis produces homocysteine, which must be remethylated to methionine for SAM regeneration, we further attempted to reinforce the methionine regeneration pathway. In this module, 5-methyltetrahydrofolate provides the methyl group required for the conversion of homocysteine to methionine, while folate metabolism supplies the corresponding one-carbon units (Fig. 4C). Therefore, the endogenous *E. coli* genes *metE*, *metF*, and *glyA* were individually or combinatorially overexpressed in TMY20 to modulate methionine-cycle regeneration. The resulting plasmids pSA-*metE*, pSA-*metF*, pSA-*glyA*, and pSA-*metE-metF-glyA* were introduced into TMY20, generating strains TMY21, TMY22, TMY23, and TMY24, respectively.

Fermentation analysis showed that expression of SAH1 increased vanillyl alcohol production to 1.74 g/L, suggesting that enhanced SAH turnover may have facilitated COMT-mediated methylation are consistent with improved methyl-donor recycling. However, individual overexpression of *metE*, *metF*, or *glyA* decreased vanillyl alcohol production to 0.95 g/L, 1.11 g/L, and 0.74 g/L, respectively. In contrast, co-expression of *metE*, *metF*, and *glyA* further increased vanillyl alcohol production to 1.95 g/L (Fig. 4D). These results suggest that partial perturbation of the methionine regeneration pathway may disrupt metabolic balance, whereas coordinated reinforcement of *metE*, *metF*, and *glyA* provides a more effective module for supporting SAM regeneration and sustaining methylation-dependent vanillyl alcohol biosynthesis.

### 3.3 Engineering vanillin accumulation and glucosylation for vanillin glucoside production

**Fig. 5:**
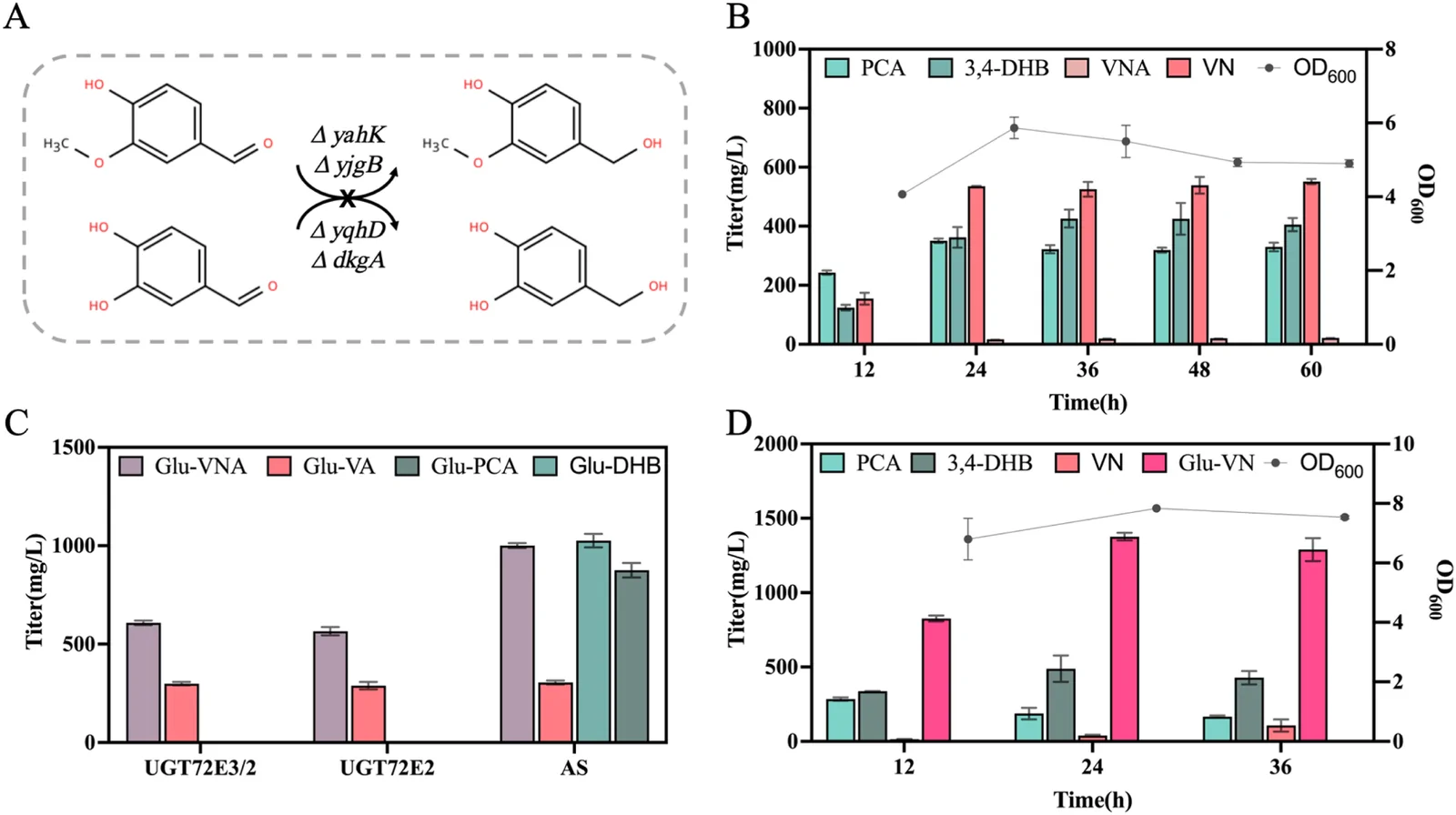
Strain construction for *de novo* biosynthesis of vanillin and vanillin glucoside. (A) Vanillin accumulation via knockout of key genes; (B) *De novo* biosynthesis of vanillin; (C) Glycosyltransferase screening; (D) *De novo* biosynthesis of vanillin glucoside.

Considering the cytotoxicity and metabolic instability of vanillin in *Escherichia coli*, vanillyl alcohol was initially selected as a less toxic and more stable target product for establishing and optimizing the methylation module. After reinforcement of the SAM cycle substantially improved vanillyl alcohol production, the optimized methylation module was further applied to redirect the pathway toward vanillin biosynthesis. *E. coli* contains multiple endogenous reductases capable of converting aromatic aldehydes into their corresponding alcohols. Among them, the reductases encoded by *yqhD*, *yjgB*, *dkgA*, and *yahK* have been reported to exhibit relatively high activity toward vanillin and are considered the major enzymes responsible for the reduction of vanillin to vanillyl alcohol (Fig. 5A) [38]. In this study, the vanillyl alcohol-producing chassis strain TMY20 was used as the starting strain for vanillin production. The endogenous reductase genes *yqhD*, *yjgB*, *dkgA*, and *yahK* were deleted by CRISPR-Cas9-mediated genome editing. In addition, the heterologous alcohol dehydrogenase gene *ADH6*, which had previously been integrated into the genome for vanillyl alcohol biosynthesis, was also removed, yielding strain TMY25. Fermentation analysis showed that deletion of these reduction-related genes redirected product formation from vanillyl alcohol toward vanillin. Vanillin production increased to 551.6 mg/L, whereas vanillyl alcohol accumulation decreased to only 22.1 mg/L (Fig. 5B). These results indicate that removal of the major endogenous reductases together with *ADH6* largely blocked the reductive conversion of vanillin to vanillyl alcohol, thereby enabling *de novo* accumulation of vanillin.

During fermentation, it was also observed that the OD_600_ of the vanillin-producing strain decreased to 5.8 relative to the parental strain, indicating that the cytotoxicity of vanillin itself, as well as its precursor 3,4-DHB, inhibited cell growth. This further confirms that product toxicity is a major bottleneck limiting further improvement in vanillin production. Glycosylation is an important strategy for reducing the toxicity of natural compounds. To alleviate the antimicrobial effect of high concentrations of vanillin, three glycosyltransferases with potential catalytic activity toward vanillin were selected. The *Arabidopsis thaliana*-derived glycosyltransferase UGT72E2 catalyzes the formation of coniferyl alcohol-4-*O*-β-d-glucosyl from coniferyl alcohol [39], while the closely related UGT72E3/2 has been reported to exhibit high catalytic activity for sinapyl alcohol-4-*O*-glucoside biosynthesis [40]. The arbutin synthase (AS) from *Rauvolfia serpentina* has been reported to efficiently catalyze the conversion of hydroquinone to arbutin [14] Considering that the above glycosyltransferases specifically catalyze glycosylation of the phenolic hydroxyl group at the 4-position, and that their substrates share certain structural similarities with vanillin, these three enzymes were selected for further evaluation. Plasmids p ET-UGT72E3/2, p ET-UGT72E2 and pET-AS were constructed under the control of PLlac O1 promoter and transformed into strain Lab01BW25113, respectively. Strains TMY26, TMY27 and TMY28 were obtained and substrate addition experiments were performed using vanillin alcohol, vanillin, 3,4-DHBA and PCA. The fermentation results showed that the three glycosyltransferases could catalyze the glycosylation of vanillin and produce 293.2 mg/L, 295.3 mg/L and 302.2 mg/L vanillin glucoside (denoted as Glu-VA), respectively, after adding vanillin and biotransforming for 6 h (Fig. 5C). However, AS exhibited broader substrate promiscuity and also showed catalytic activity toward certain pathway intermediates. Subsequently, pET-UGT72E3/2 was introduced into the previously constructed TMY25, generating strain TMY29. As shown in Fig. 5D, overexpression of the glycosyltransferase improved cell growth in the engineered strain: compared with strain TMY25, which exhibited an OD_600_ of 5.8, the OD_600_ of TMY29 increased to 7.1, indicating that glycosyltransferase expression alleviated vanillin-associated cytotoxicity. Vanillin glucoside production was successfully detected in TMY29, reaching a maximum titer of 1.37 g/L, equivalent to 660 mg/L vanillin on a molar basis, while the residual vanillin in the culture broth decreased to 38.9 mg/L. These results indicate that UGT72E3/2 efficiently catalyzed vanillin glycosylation, converting most of the vanillin into vanillin glucoside. In addition, no glycosylated products derived from other pathway precursors were detected, suggesting that UGT72E3/2 exhibits high substrate specificity toward vanillin and does not glycosylate other intracellular metabolites. This substrate specificity likely prevented unnecessary dissipation of metabolic flux and thereby ensured efficient biosynthesis of vanillin glucoside.

### 3.4 Fed-batch fermentation for enhanced vanillin glucoside production

**Fig. 6:**
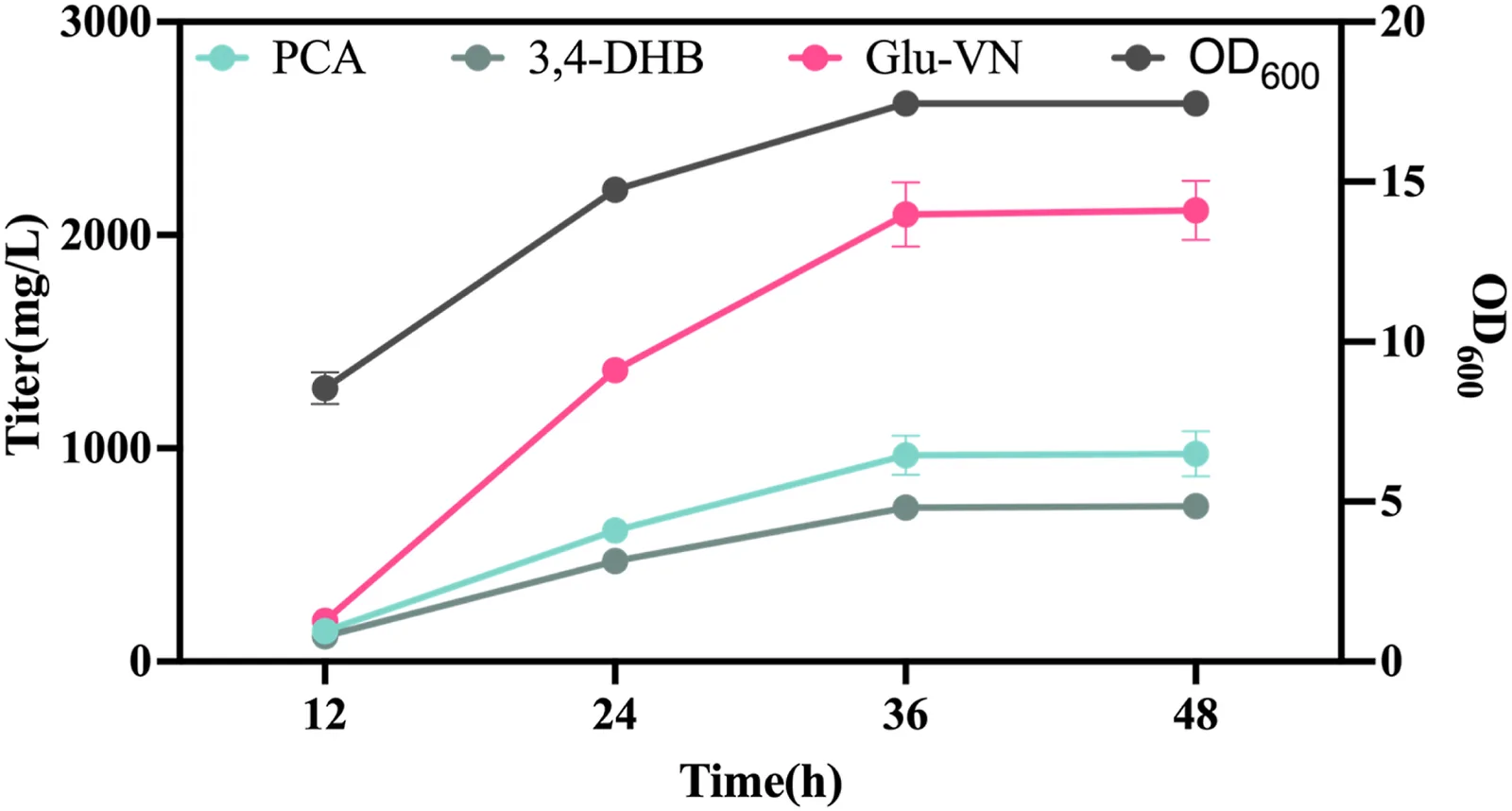
*De novo* synthesis of vanillin glucoside with 3-L fed-batch fermenter.

To evaluate the scale-up potential of the engineered strain, fed-batch fermentation for vanillin glucoside production was performed in a 3-L bioreactor using strain TMY29. The culture was maintained at 33°C, and IPTG was added to induce pathway gene expression when the OD_600_ reached approximately 6. After induction, the cells continued to grow, and the OD_600_ reached approximately 17 at 48 h. Vanillin glucoside accumulation followed a similar trend to cell growth, gradually increasing during fermentation and reaching a maximum titer of 2.12 g/L at 48 h (Fig. 6). These results indicate that TMY29 was able to produce vanillin glucoside under bioreactor conditions, demonstrating the feasibility of scale-up fermentation. However, cell growth remained relatively limited during the fed-batch process. Although glycosylation of vanillin may partially alleviate vanillin-associated toxicity, the accumulation of upstream aldehyde intermediates, particularly 3,4-DHB, may still impose cytotoxic stress on the host strain and contribute to impaired growth. Therefore, further improvement of precursor conversion, methylation efficiency, UDP-glucose supply, and feeding strategy will be required to enhance strain robustness and achieve higher vanillin glucoside titers in future scale-up processes.

## 4 Discussion

In this study, we established a modular biosynthetic platform in *Escherichia coli* for the *de novo* production of vanillyl alcohol, vanillin, and vanillin glucoside from glycerol. The results highlight that efficient production of methoxy-substituted aromatic compounds depends not only on pathway reconstruction, but also on coordinated control of cofactor supply, competing endogenous reactions, and product detoxification. In particular, the improved performance of the engineered strain after chromosomal integration of *ADH6* and *COMT* indicates that stable expression of key pathway enzymes is critical for maintaining metabolic flux. More importantly, the subsequent reinforcement of the SAM regeneration cycle markedly increased vanillyl alcohol accumulation, suggesting that methyltransferase-catalyzed steps are strongly constrained by intracellular methyl-donor availability rather than by enzyme expression alone. This observation is consistent with previous studies showing that SAM limitation often represents a major bottleneck in O-methylation pathways, and that strengthening SAM recycling can be more effective than simply overexpressing methyltransferases.

The accumulation of vanillin was further improved by deletion of endogenous aldehyde-reducing enzymes, including *yqhD, yjgB, dkgA, yahK* and *ADH6*. The results highlight that efficient production of methoxy-substituted aromatic compounds depends not only on pathway reconstruction, but also on coordinated control of cofactor supply, competing endogenous reactions, and product detoxification. In particular, the improved performance of the engineered strain after chromosomal integration of *ADH6* and *COMT* indicates that stable expression of key pathway enzymes is critical for maintaining metabolic flux. More importantly, the subsequent reinforcement of the SAM regeneration cycle markedly increased vanillyl alcohol accumulation, suggesting that methyltransferase-catalyzed steps are strongly constrained by intracellular methyl-donor availability rather than by enzyme expression alone. This observation is consistent with previous studies showing that SAM limitation often represents a major bottleneck in O-methylation pathways, and that strengthening SAM recycling can be more effective than simply overexpressing methyltransferases.

Compared with previous studies on microbial vanillin production, this work provides a more integrated design strategy that links precursor supply, cofactor regeneration, competing pathway suppression, and downstream modification. Whereas earlier approaches often focused on individual bottlenecks, such as enhancing a single enzyme or blocking one reduction route, our study demonstrates that high-level production requires simultaneous optimization of multiple layers of metabolic control. The achieved titers of vanillyl alcohol, vanillin, and vanillin glucoside therefore reflect not only enzyme engineering but also effective system-level pathway balancing. In this sense, the platform developed here offers a transferable framework for the biosynthesis of other value-added phenolic compounds.

From an industrial perspective, the use of glycerol as the carbon source further enhances the practical relevance of this process. Glycerol is an inexpensive and renewable feedstock, making the developed pathway attractive for sustainable flavor and fragrance manufacturing. Moreover, vanillin glucoside may have additional application potential as a more stable and less toxic storage or transport form of vanillin, which could expand its utility in food, cosmetic, and functional ingredient markets. Overall, this study advances the microbial synthesis of vanillin-derived compounds and provides a foundation for scalable and modular production of natural aromatic glycosides.

## CRediT authorship contribution statement

**Mingyu Tian:** Writing – review & editing, Writing – original draft, Investigation, Formal analysis, Data curation. **Jinyi Li:** Visualization, Resources, Project administration, Methodology, Data curation, Conceptualization. **Yong Du:** Supervision, Investigation, Formal analysis, Data curation. **Xiaolin Shen:** Writing – review & editing. **Jia Wang:** Writing – review & editing. **Xinxiao Sun:** Writing – review & editing, Supervision, Conceptualization. **Qipeng Yuan:** Writing – review & editing.

## Declaration of competing interest

The authors declare that they have no known competing financial interests or personal relationships that could have appeared to influence the work reported in this paper.
