# Supplementary material for: Biosynthesis of vanillin glucoside in Escherichia coli
Source: Synth Syst Biotechnol. 2026 Sep 8;18:96–103. doi: 10.1016/j.synbio.2026.08.008 (PMC13578468; doi:10.1016/j.synbio.2026.08.008)
Supplement: Supplementary file 1 [file mmc1.docx]

**Biosynthesis of Vanillin Glucoside in *Escherichia coli***

Mingyu Tian^a^, Jinyi Li^a^, Yong Du^a^, Xiaolin Shen^a^, Jia Wang^a^, Xinxiao Sun^a^* and Qipeng Yuan^a^

^a^State Key Laboratory of Chemical Resource Engineering, Beijing University of Chemical Technology, Beijing, China 100029

**Table S1** Strains used in this study

| Strain | Genotype | Plasmids |
| --- | --- | --- |
| Lab01 | BW25113 | / |
| Lab02 | BW25113*ΔpykA ΔpykF*  *yghX*::P50trc-*aroG^fbr^*, *mbhA*::P10trc-*quiC* | / |
| TMY01 | Lab02 | pZE-AtCOMT-V314I pCS-car-sfp pSA-*ADH6* |
| TMY02 | Lab02 *gapC*::P10trc-*ADH6* | pZE-AtCOMT-V314I pCS-car-sfp |
| TMY03 | Lab02 *gapC*::Plpp2.0-*ADH6* | pZE-AtCOMT-V314I pCS-car-sfp |
| TMY04 | Lab02 *gapC*::Plpp4.0-*ADH6* | pZE-AtCOMT-V314I pCS-car-sfp |
| TMY05 | TMY04 *ylbE*::Plpp4.0-AtCOMT-V314I | pCS-car-sfp |
| TMY06 | TMY05 *ilvG*::Plpp4.0-AtCOMT-V314I | pCS-car-sfp |
| TMY07 | TMY06 *yncI*::Plpp4.0-AtCOMT-V314I | pCS-car-sfp |
| TMY08 | TMY07 *yeeL*::Plpp4.0-AtCOMT-V314I | pCS-car-sfp |
| TMY09 | TMY08 *ycgH*::Plpp4.0-AtCOMT-V314I | pCS-car-sfp |
| TMY10 | TMY09 *yeeP*::Plpp4.0-AtCOMT-V314I | pCS-car-sfp |
| TMY11 | TMY09 *yeeP*::Plpp4.0-AtCOMT-V314I | pCS-car-sfp-AtCOMT-V314I |
| TMY12 | TMY09 *yeeP*::Plpp4.0-AtCOMT-V314I | pCS-P10trc-car-sfp-AtCOMT-V314I |
| TMY13 | TMY09 *yeeP*::Plpp4.0-AtCOMT-V314I | pCS-P30trc-car-sfp-AtCOMT-V314I |
| TMY14 | TMY09 *yeeP*::Plpp4.0-AtCOMT-V314I | pCS-P50trc-car-sfp-AtCOMT-V314I |
| TMY15 | TMY09 *yeeP*::Plpp4.0-AtCOMT-V314I | pCS-P10trc-car-sfp-AtCOMT-V314I pSA-*metK* |
| TMY16 | TMY09 *yeeP*::Plpp4.0-AtCOMT-V314I | pCS-P10trc-car-sfp-AtCOMT-V314I pSA-*SAM2* |
| TMY17 | TMY12 *speD*::P10trc-*SAM2* | pCS-P10trc-car-sfp-AtCOMT-V314I |
| TMY18 | TMY12 *speD*::P30trc-*SAM2* | pCS-P10trc-car-sfp-AtCOMT-V314I |
| TMY19 | TMY12 *speD*::P50trc-*SAM2* | pCS-P10trc-car-sfp-AtCOMT-V314I |
| TMY20 | TMY18 *tyrR*::P10trc-*SAH1* | pCS-P10trc-car-sfp-AtCOMT-V314I |
| TMY21 | TMY20 | pCS-P10trc-car-sfp-AtCOMT-V314I pSA-*metE* |
| TMY22 | TMY20 | pCS-P10trc-car-sfp-AtCOMT-V314I pSA-*metF* |
| TMY23 | TMY20 | pCS-P10trc-car-sfp-AtCOMT-V314I pSA-*glyA* |
| TMY24 | TMY20 | pCS-P10trc-car-sfp-AtCOMT-V314I pSA-*EFA* |
| TMY25 | TMY20 *ΔyqhD ΔdkgA ΔyahK*  *ΔADH6 ΔyjgB* | pCS-P10trc-car-sfp-AtCOMT-V314I |
| TMY26 | Lab02 | pET-UGT72E2 |
| TMY27 | Lab02 | pET-UGT72E3/2 |
| TMY28 | Lab02 | pET-AS |
| TMY29 | TMY25 | pET-UGT72E3/2 pCS-P10trc-car-sfp-AtCOMT-V314I |

**Table S2** Plasmids used in this study

| Plasmid | Description | Source |
| --- | --- | --- |
| pZE-AtCOMT-V314I | pZE12-luc, single mutation of V314I based on *COMT* from *Arabidopsis thaliana*, *Amp*^R^ | Lab stock |
| pCS-car-sfp | pCS27, *car* from *M.marinum*, *sfp* from *B.subtilis*, Kan^R^ | Lab stock |
| pSA-*ADH6* | pSA74, *ADH6* from *Saccharomyces cerevisiae*, Cm^R^ | Lab stock |
| pCS-CSC | the AtCOMT-V314I gene was inserted into the second reading frame based on pCS-car-sfp, Kan^R^ | This study |
| pCS-P10trc-CSC | pCS-CSC, P10trc promoter, *Kan^R^* | This study |
| pCS-P30trc-CSC | pCS-CSC, P30trc promoter, *Kan^R^* | This study |
| pCS-P50trc-CSC | pCS-CSC, P50trc promoter, *Kan^R^* | This study |
| pSA-*SAM2* | pSA74, *SAM2* from *S. cerevisiae*, *Cm^R^* | This study |
| pSA-*SAH1* | pSA74, *SAH1* from *S. cerevisiae*, *Cm^R^* | This study |
| pSA-*metE* | pSA74, *metE* from *E. coli*, *Cm^R^* | This study |
| pSA-*metF* | pSA74, *metF* from *E. coli*, *Cm^R^* | This study |
| pSA-*glyA* | pSA74, *glyA* from *E. coli*, *Cm^R^* | This study |
| pSA-EFA | pSA74, *metE metF* and *glyA* from *E. coli*, *Cm^R^* | This study |
| pET-UGT72E2 | pET-lac, *UGT72E2* from *A. thaliana*, *Amp^R^* | This study |
| pET-UGT72E3/2 | pET-lac, *UGT72E3/2* from *A. thaliana*, Amp^R^ | This study |
| pET-AS | pET-lac, *AS* from *Rauvolfia serpentina*, *Amp^R^* | This study |

**Table S3** The gene accession numbers

| Gene | NCBI accession number |
| --- | --- |
| *aroG^fbr^* | NC_000913.3 |
| *quiC* | NC_021505.1 |
| *AtCOMT* | NC_003076.8 |
| *car* | NZ_HG917972.2 |
| *sfp* | NC_000964.3 |
| *ADH6* | NC_001145.3 |
| *SAM2* | NC_00113610 |
| *SAH1* | NC_001137.3 |
| *metE* | NC_000913.3 |
| *metF* | NC_000913.3 |
| *glyA* | NC_000913.3 |
| *UGT72E2* | NC_003076.8 |
